# Targeting ferroptosis as a promising therapeutic strategy to treat cardiomyopathy

**DOI:** 10.3389/fphar.2023.1146651

**Published:** 2023-04-13

**Authors:** Huiyan Sun, Dandan Chen, Wenjing Xin, Lixue Ren, Qiang LI, Xuchen Han

**Affiliations:** ^1^ Health Science Center, Chifeng University, Chifeng, China; ^2^ Key Laboratory of Human Genetic Diseases in Inner Mongolia, Chifeng, China; ^3^ Department of Endocrinology, The Affiliated Hospital of Chifeng University, Chifeng, China; ^4^ Chifeng Clinical Medical College, Inner Mongolia Minzu University, Tongliao, China; ^5^ Department of Neurology, The Affiliated Hospital of Chifeng University, Chifeng, China; ^6^ Department of Cardiology, The Affiliated Hospital of Chifeng University, Chifeng, China

**Keywords:** cardiomyopathies, ferroptosis, ferroptosis inhibitor, treatment, bioactive compounds

## Abstract

Cardiomyopathies are a clinically heterogeneous group of cardiac diseases characterized by heart muscle damage, resulting in myocardium disorders, diminished cardiac function, heart failure, and even sudden cardiac death. The molecular mechanisms underlying the damage to cardiomyocytes remain unclear. Emerging studies have demonstrated that ferroptosis, an iron-dependent non-apoptotic regulated form of cell death characterized by iron dyshomeostasis and lipid peroxidation, contributes to the development of ischemic cardiomyopathy, diabetic cardiomyopathy, doxorubicin-induced cardiomyopathy, and septic cardiomyopathy. Numerous compounds have exerted potential therapeutic effects on cardiomyopathies by inhibiting ferroptosis. In this review, we summarize the core mechanism by which ferroptosis leads to the development of these cardiomyopathies. We emphasize the emerging types of therapeutic compounds that can inhibit ferroptosis and delineate their beneficial effects in treating cardiomyopathies. This review suggests that inhibiting ferroptosis pharmacologically may be a potential therapeutic strategy for cardiomyopathy treatment.

## Introduction

Cardiomyopathies are a clinically heterogeneous group of cardiac diseases characterized by heart muscle damage, causing cardiac muscle or myocardium disorders, diminished cardiac function, heart failure, and even sudden cardiac death (Franz et al., 2001; Schultheiss et al., 2019; Li D. et al., 2022). Cardiomyopathies are often related to electrical or mechanical dysfunction, frequently with a genetic origin or etiology (Maron et al., 2006). The 2006 American Heart Association classification categorizes and groups cardiomyopathy into primary or secondary categories (Maron et al., 2006). In primary categories (genetic, mixed, or acquired), the disease process is solely or predominantly confined to the heart. Secondary cardiomyopathies (i.e., dilated, hypertrophic, and restrictive cardiomyopathy) result from systemic conditions, i.e., cardiac involvement occurs as a part of systemic conditions (Brieler et al., 2017; Li T. et al., 2022). Researchers have divided the secondary causes of cardiomyopathy into various categories, including infectious, toxic, ischemic, metabolic, autoimmunogenic, and neuromuscular categories. The burden of ischemic cardiomyopathy (ICM), diabetic cardiomyopathy (DCM), doxorubicin-induced cardiomyopathy (DICM), and septic cardiomyopathy (SCM) is increasing in nearly all countries. The basic pathological mechanism of these cardiomyopathies (ICM, DCM, DICM and SCM) is cell death in cardiomyocytes. The pathogenesis and molecular mechanisms underlying these cardiomyopathies are poorly understood, warranting further investigation (Gilgenkrantz et al., 2021). Therefore, it is important to acquire insights into their pathogenesis to achieve the appropriate management and treatment of these disorders, thus providing support for protecting cardiac function.

In the past decades, ferroptosis, a non-apoptotic iron-dependent and peroxidation-driven regulated cell death (RCD) mechanism, has been rapidly acquiring attention in cardiomyopathies. Novel studies have explored the role of ferroptosis in DICM and ICM in murine models of cardiomyopathy (Conrad and Proneth, 2019; Fang et al., 2019), which demonstrated an association between ferroptosis and cardiac cell death induced by iron overload *in vivo*. Thereafter, several studies have revealed that ferroptosis plays a vital role in the pathogenesis of cardiomyopathy (Li D. et al., 2022). Meanwhile, certain compounds exert their therapeutic effects on experimental cardiomyopathy models by inhibiting ferroptosis.

In this review, we summarize the core mechanism by which ferroptosis leads to the genesis of cardiomyopathies. We focus on the emerging variety of therapeutic compounds that can inhibit ferroptosis and delineate their beneficial effects for treating cardiomyopathies. This review indicates that inhibiting ferroptosis pharmacologically may be a promising therapeutic strategy for treating cardiomyopathies.

### Core molecular mechanisms underlying ferroptosis

Ferroptosis is an iron-dependent, oxidative form of non-apoptotic RCD, characterized by the iron-dependent oxidative modification of phospholipid membranes (Dixon et al., 2012). A delicate imbalance between ferroptosis inducers and inhibitors dictates its execution and induction. The inhibition of the solute carrier family 7 member 11/glutathione peroxidase 4 (SLC7A11/GPX4) antioxidant system and free iron accumulation are two key signals for inducing ferroptosis (Chen H. Y. et al., 2021). When the levels of iron-dependent ROS and lethal lipid peroxide (LPO), the two promoting factors of ferroptosis, substantially surpass the antiferroptotic capacity of ferroptosis defense systems, peroxidated phospholipid polyunsaturated fatty acids (PUFA-PL-OOH) accumulate on cellular membranes and induce its rupture, eventually resulting in ferroptosis (Lei et al., 2022). Phospholipid polyunsaturated fatty acids (PUFA-PLs) have an intrinsic susceptibility to peroxidation chemistry, which makes them the primary substrates for LPO (Hadian and Stockwell, 2020). Acyl-coenzyme A synthetase long chain family member 4 (ACSL4) catalyzes the addition of coenzyme A (CoA) to the long-chain polyunsaturated bonds of arachidonic acid (AA), causing PUFA esterification to form phospholipids. Following the activation of ACSL4, lysophosphatidylcholine acyltransferase 3 (LPCAT3) inserts acyl groups into lysophospholipids and incorporates free PUFAs into phospholipids (PL), participating in ferroptotic lipid signaling. Under the catalysis of oxidase and bioactive iron, PUFA-PLs in the membrane can be converted to phospholipid peroxides by both non-enzymatic Fenton reactions and enzymatic LPO reactions (Chen et al., 2021b; Liang et al., 2022). Iron serves as an essential cofactor for arachidonate lipoxygenases (ALOXs) and cytochrome P450 oxidoreductase (POR) to initiate the non-enzymatic Fenton reaction. ALOXs and POR promote lipid peroxidation. In enzymatic LPO, ACSL4 catalyzes the ligation of free PUFAs [such as AA and adrenic acid (AdA)] with CoA to generate PUFA-CoAs, which include AA-CoA or AdA-CoA (Dixon et al., 2015; Doll et al., 2017). Subsequently, LPCAT3 incorporates PUFA-CoAs into *p*Ls to generate PUFA-PLs, which includes AA-phosphatidylethanolamine or AdA-phosphatidylethanolamine (Dixon et al., 2015; Kagan et al., 2017). Once the PUFA-PLs incorporated lipid bilayers, the iron-dependent enzymes (such as POR and ALOXs) and labile iron use O_2_ to perform a peroxidation reaction, generating peroxidated PUFA-PL or polyunsaturated-fatty-acid-containing -phospholipid hydroperoxides (PUFA-PL-OOH) (Hadian and Stockwell, 2020; Zou et al., 2020). Other membrane electron transfer proteins, particularly the NADPH oxidases, are also involved in ferroptosis by contributing to ROS production for LPO (Xie et al., 2017). LPO and its secondary products, namely, malondialdehyde (MDA) and 4-hydroxynonenal (4-HNE), cause pore formation in the lipid bilayers, eventually resulting in cell death and ferroptosis (Tang and Kroemer, 2020). Ferroptosis has acquired substantial attention in cardiomyopathy research. Further, it plays a vital role in the pathogenesis of cardiomyopathies, such as ICM, DCM, DICM, and SCM. Therapeutic strategies targeting ferroptosis may facilitate the treatment of these cardiomyopathies.

## Ferroptosis in cardiomyopathy

### Ferroptosis in ischemic cardiomyopathy

Ischemic cardiomyopathy (ICM) principally results from long-term ischemia/hypoxia within coronary atherosclerosis; it impairs the systolic or diastolic function of the heart. ICM represents the leading cause of heart failure (HF) worldwide (Chang et al., 2022; Del Buono et al., 2022). Further, it leads to numerous phenotypic changes, such as myocardial remodeling and HF.

Metabolic, neurohumoral, and inflammatory alterations are involved in the pathophysiological mechanisms underlying ICM, leading to hypertrophy in cardiomyocytes, fibrosis, calcium dyshomeostasis, inflammation, oxidative stress, and even cardiomyocyte death (Cabac-Pogorevici et al., 2020). Ischemic heart disease is a major contributor to the global disease burden and constitutes the leading cause of global mortality worldwide. Acute myocardial infarction (AMI) resulting from reduced oxygen supply causes initial damage to the cardiac tissues, thus making it the primary cause of disability and mortality. Myocardial reperfusion strategies and reoxygenation are effective and the preferred treatment for AMI; non-etheless, reperfusion inevitably triggers the cell death of cardiomyocytes, increases the infarct size, and worsens the condition, which is referred to as myocardial ischemia-reperfusion injury (MIRI) (Ibáñez et al., 2015). MIRI leads to oxidative stress and energy metabolism disturbances, among other issues (Li D. et al., 2021). Therefore, understanding the mechanisms of MIRI is essential for attenuating the triggers of cardiomyocyte cell death and preventing left ventricular remodeling and HF.

**FIGURE 1:**
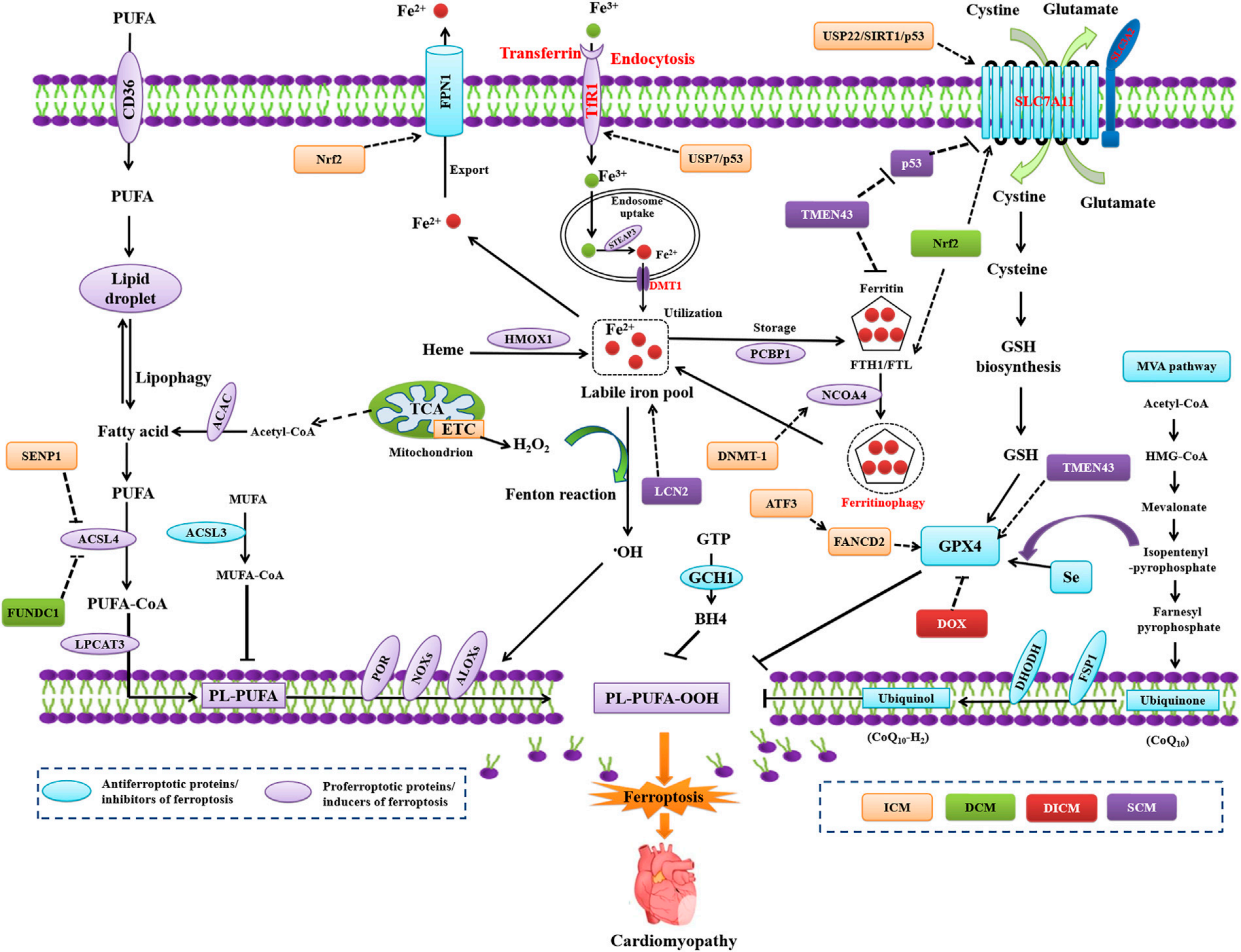
Regulation of ferroptosis in model in ICM, DCM, DICM, and SCM. ATF3, activating transcription factor 3; DNMT-1, DNA (cytosine-5) -methyltransferase 1; FUNDC1, FUN14 domain containing 1; LCN2, neutrophil-derived lipocalin-2; SENP1, sentrin-specific protease 1; TMEM43, transmembrane protein 43; USP7, ubiquitin-specific protease 7.

A novel study reported on the role of ferroptosis in ischemia/reperfusion (I/R)-induced cardiomyopathy in murine models (Fang et al., 2019), which established an *in vivo* correlation between ferroptosis and cardiac cell death (Conrad and Proneth, 2019). Thereafter, emerging studies delved into the pathophysiological role of ferroptosis in the development of MIRI and ICM (Figure 1). Numerous molecular mechanisms and pathways are related to the genesis of MIRI, including iron homeostasis imbalance, lipid peroxidation, and redox homeostasis imbalance. Since the introduction of ferroptosis in 2012, researchers have revisited the role of iron homeostasis imbalance, lipid peroxidation, or glutathione metabolism disorder in MIRI, thus proposing that ferroptosis participates in MIRI pathogenesis. Among all types of organ ischemia/reperfusion injury (IRI), the role of ferroptosis in the pathogenesis of MIRI has been the most extensively studied.

#### Role of dysregulation of iron metabolism in MIRI

The accumulation of iron, a core characteristic of ferroptosis, plays a pathogenic role in AMI and MIRI. Excessive iron is transported into the cardiomyocytes, thus predisposing them to undergo ferroptosis by the Fenton reaction and ROS generation after I/R (Li J. Y. et al., 2021). Ferroptosis predominantly occurs in the reperfusion phase of cardiac tissues, characterized by a gradual increase in the ACSL4, Fe^2+^, and MDA levels, along with decreased levels of GPX4 (Tang et al., 2021a). Cardiomyocytes are vulnerable to the dysregulation of iron homeostasis, which is central to MIRI through different pathways to increase the iron content. The heart utilizes several iron uptake transport systems, including L-type (LTCC) or T-type (TTCC) voltage-dependent Ca^2+^ channels, transferrin (TF) receptor (TfR1), and divalent metal transporter (DMT1) (Lillo-Moya et al., 2021).

Iron enters the cardiomyocytes principally through TfR1 as TF or through LTCC as non-TF-bound iron, TTCC, and DMT1. During MIRI, the intracellular iron-storing protein, the degraded ferritin to release iron and perform iron-mediated Fenton reaction, resulting in oxidative damage to cardiomyocytes and loss of cardiac function. Studies have demonstrated excessive iron accumulation in the myocardial scar in mice MIRI models (Baba et al., 2018; Fang et al., 2019), thereby suggesting iron overload as a primary characteristic of ferroptosis. The ferroptosis inhibitor ferostatin-1 (Fer-1) or iron chelator dexrazoxane (DXZ) inhibits cardiac remodeling and fibrosis induced by IRI (Fang et al., 2019). Increased cellular iron content exists in IRI mice, apart from decreased activities of GPX4 and ferritin heavy chain-1 (FTH1) as well as decreased glutathione (GSH) levels in the cardiac issue after MIRI (Chen et al., 2021c). Moreover, the ubiquitin-specific protease 7 (USP7)/p53 pathway activates TfR1 to exacerbate cardiomyocyte ferroptosis in subsequent I/R (Tang et al., 2021a). The pharmacological inhibition of USP7 results in increased p53 activity and decreased TfR1, thus leading to decreased ferroptosis and MIRI (Tang et al., 2021b). Therefore, the pharmacological inhibition of TfR1 activity may inhibit ferroptosis in MIRI.

Nuclear receptor coactivator 4 (NCOA4)-mediated autophagy, i.e., ferritinophagy, causes I/R-induced ferroptosis. The activation of ferritinophagy degrades ferritin and increases the availability of iron in the cells (Lin et al., 2020). FTH1 binds to NCOA4 that functions as a selective autophagy receptor; it transports intracellular ferritin to the autophagy lysosomes and releases free iron (Mancias et al., 2014). Ferritinophagy induces ferroptosis through iron homeostasis regulation and ROS production in cells (Gao et al., 2016; Hou et al., 2016; Liu H. et al., 2022). Inhibiting DNA (cytosine-5)-methyltransferase 1 attenuates ferroptosis by impeding NCOA4 -mediated ferritinophagy in diabetic MIRI (Li J. Y. et al., 2021). The iron exporter ferroportin 1 (FPN1) plays a vital role in regulating iron homeostasis. Hepcidin, an iron-regulating hormone, mediates the internalization and degradation of FPN1, which maintains cardiac iron homeostasis (Lakhal-Littleton et al., 2015). Nuclear factor erythroid 2–related factor 2 (Nrf2) transcriptionally regulates FPN1. Nrf2/FPN1 signaling activation can alleviate MIRI by inhibiting ferroptosis (Tian H. et al., 2021). FTH deletion in the myocardium upregulates heme oxygenase-1 (HO-1) among other antiferroptotic proteins, which induces SLC7A11 and finally inhibits IRI-induced ferroptosis, thereby maintaining the function of myocardium (Machado et al., 2022).

#### Role of LPO in MIRI

Deferoxamine therapy decreases myocardial injury by inhibiting ferroptosis in I/R-induced rat hearts. The specific redox reactions of PUFA-PLs in ischemia-induced cardiomyocytes initiate oxidative damage in the reperfusion phase. ALOX15 induction by ischemia/hypoxia initiates the oxidation of PUFA-PLs (particularly PUFA-PE) and results in cardiomyocyte ferroptosis. Further, ALOX15 ablation in mice confers resistance to PUFA-dependent ischemia-induced cardiac injury (Ma X. et al., 2022). The overexpression of activating transcription factor 3 (ATF3) inhibits the classical ferroptosis activators ras-selective lethal small molecule 3 and erastin-induced ferroptosis in cardiomyocytes. ATF3 expression increases in the early phase of reperfusion, whereas its ablation significantly aggravates IRI. The binding of ATF3 to the transcriptional start site of the FA complementation group D2 can enhance its promoter activity, thereby exerting cardioprotective effects against H/R injury through an antiferroptosis mechanism (Liu M. Z. et al., 2022). Bai and colleagues have demonstrated that SENP1 expression is upregulated by hypoxia, which protects cardiomyocytes against ferroptosis through deSUMOylating hypoxia-inducible factor-1α and ACSL4 (Bai et al., 2021).

#### Role of SLC7A11/GPX4 axis inhibition in MIRI

Increased levels of ACSL4, Fe^2+^, and MDA, along with decreased GPX4 levels, are observed in the myocardium after MIRI (Tang et al., 2021a). The inhibition of the GSH-generation pathway, either iron chelation or glutaminolysis, could alleviate IRI by blocking ferroptosis (Gao et al., 2015). A specific ferroptosis inhibitor suitable for animal models, i.e., liproxstatin 1, can protect the mouse myocardium against IRI by decreasing voltage-dependent anion-selective channel protein 1 levels and upregulating GPX4 levels (Feng et al., 2019). The expression of USP22, SIRT1, and SLC7A11 is inhibited after IRI injury, whereas p53 is highly expressed in the myocardial tissues. Conversely, the overexpression of USP22, SIRT1, or SLC7A11 reduces the degree of IRI injury by inhibiting ferroptosis and improves the viability of cardiomyocytes (Ma et al., 2020).

### Ferroptosis in diabetic cardiomyopathy

DCM, a specific form of cardiomyopathy independent of hypertension and coronary artery disease (Tan et al., 2020), is caused by diabetes mellitus (DM)-associated dysregulated glucose and lipid metabolism (Tan et al., 2020). DM increases oxidative stress and activates multiple inflammatory pathways, leading to cellular injury, cardiac remodeling, and systolic and diastolic dysfunction (Tan et al., 2020; Khan et al., 2021). The eventual outcome is cardiomyocyte cell death. The clinical features and pathogenesis of DCM have been well-characterized in the past 4 decades; however, its effective therapeutic regimen is still limited, thus suggesting the need to explore novel mechanisms underlying DCM development. Ferroptosis may be associated with the pathological progression of DCM (Chen et al., 2020; Wei LY. et al., 2022; Wei Z. et al., 2022). Ferroptosis plays a role in DM (Behring et al., 2014; Bruni et al., 2018; Lutchmansingh et al., 2018; Shu et al., 2019; Krümmel et al., 2021) (Figure 1). A novel study reported on the role of ferroptosis in the heart of diabetic mice in 2022, thus demonstrating that Nrf2 activation attenuates ferroptosis by upregulating SLC7A11 and ferritin levels (Wang D. et al., 2022). GPX4 can inhibit DCM in GPX4 transgenic mouse models (Baseler et al., 2013).

The ablation of cluster of differentiation 74 (CD74; a receptor for the regulatory cytokine macrophage migration inhibitory factor) prevents DM-evoked and oxidative stress. Ferroptosis inhibitors preserve the cardiomyocyte function and inhibit LPO induced by the high glucose/high fat (HGHF) challenge *in vitro*. Recombinant MIF mimics HGHF-induced LPO and depletes GSH and ferroptosis. Conversely, MIF inhibitors reverse these effects mediated by recombinant MIF. Taken together, CD74 ablation rescues DCM by inhibiting ferroptosis, thus indicating CD74 as a promoter of ferroptosis (Chen H. et al., 2022). FUN14 domain-containing 1 (FUNDC1) insufficiency sensitizes DCM through ACSL4-mediated ferroptosis, thus indicating FUNDC1 as an inhibitor of ferroptosis (Pei et al., 2021). Further, long non-coding RNAs (LncRNAs) regulate ferroptosis in DCM. The lncRNA-zinc finger antisense 1 works as a competing endogenous RNA that sponges miR-150-5p and downregulates cyclin D2 (CCND2), promoting ferroptosis and DCM development (Ni J. et al., 2021). In summary, ferroptosis plays a significant role in the development of DCM. However, the molecular mechanism warrants further investigation.

### Ferroptosis in DOX-induced cardiomyopathy

Anthracyclines are the most widely used anticancer chemotherapeutic agents. However, doxorubicin (DOX) causes cardiotoxicity, resulting in DICM, thereby limiting its clinical efficacy (Herrmann, 2020; Fang et al., 2023). Ferroptosis plays an essential role in the pathogenesis of DICM (Fang et al., 2023) (Figure 1). Wang et al. have demonstrated that DOX induces heart injury and increases cardiac iron levels, lipid-derived ROS, and the biomarkers of ferroptosis (Fang et al., 2019). They presented novel evidence that the contributions of ferroptosis to DICM in DOX-treated mice and its subsequent inhibition exert cardioprotection (Fang et al., 2019). Their findings were corroborated by other studies which revealed that ferroptosis is a crucial mechanism in DICM and that acyl-CoA thioesterase 1 (ACOT1) plays a critical role during the process. Thus, they demonstrated ACOT1 as a ferroptosis inhibitor and that targeting the inhibition of ferroptosis is a strategy for DICM treatment (Liu et al., 2020). Tadokoro and colleagues have revealed that DOX inhibits GPX4 and induces LPO, thus leading to mitochondria-dependent ferroptosis in a DICM mouse model (Tadokoro et al., 2020). Further, the ferroptosis inhibitor ferrostatin-1 (Fer-1) can protect cardiomyocytes against DOX-induced cell injury (Tadokoro et al., 2020). Zhang et al. (2021) have indicated that DOX upregulates high mobility group box 1 expression, which promotes ferroptosis-associated cardiotoxicity in DOX-treated rats. Fer-1 or DXZ reverse DOX-induced ferroptosis and DICM. In summary, ferroptosis inhibition is a therapeutic target for DICM.

### Ferroptosis in septic cardiomyopathy

Sepsis is a life-threatening organ dysfunction resulting from dysregulated immune response to an infection. Seventy percent of patients with sepsis develop septic cardiomyopathy (SCM), which is the leading cause of sepsis-related morbidity and mortality (Nabzdyk et al., 2019; Hollenberg and Singer, 2021). Ferroptosis is involved in SCM (Figure 1). GSH depletion and the downregulation of GPX4 expression, as well as increased iron content and LPO levels, exist in cecal ligation and puncture-induced sepsis animal model, implying the involvement of ferroptosis in the pathogenesis of SCM (Wang et al., 2020). Dexmedetomidine exerts cardioprotective effects through ferroptosis inhibition by decreasing iron accumulation, downregulating the protein levels of HO-1, and inducing GPX4 (Wang et al., 2020). The ferroptosis inhibitors deferoxamine and Fer-1 can improve cardiac function and decrease mortality in septic mice by decreasing the levels of ferroptosis in cardiomyocytes (Li et al., 2020). These results support the hypothesis that ferroptosis is involved in the pathogenesis of sepsis-induced myocardial injury. Ferritinophagy-mediated ferroptosis plays a pathogenic role in sepsis-induced myocardial injury (Li et al., 2020). Li et al. (2020) have demonstrated that ferroptosis plays a crucial role in sepsis-induced cardiomyopathy in sepsis-related models, including a lipopolysaccharide (LPS)-induced model of septic cardiomyopathy (Li et al., 2020).

Specific regulators play a role in modulating ferroptosis and SCM. The transmembrane protein 43 (TMEM43), a transmembrane protein related to cardiomyopathy, protects against SCM by inhibiting ferroptosis in LPS-induced mice (Chen L. et al., 2022). The knockdown of TMEM43 in the heart aggravates LPS-induced cardiomyopathy, accompanied by an increased cardiac ferroptosis. TMEM43 overexpression decreases LPS-induced ferroptosis and cardiac injury by inhibiting LPO. TMEM43 silencing promotes ferroptosis and cell injury in LPS-induced rat H9c2 cardiomyocytes. TMEM43 downregulates the expression of P53 and ferritin but upregulates the levels of GPX4 and SLC7A11, thereby inhibiting LPS-induced ferroptosis. Fer-1 can ameliorate TMEM43 knockdown-induced deteriorating effects in LPS-induced cardiac injury. Taken together, TMEM43 protects against SCM by inhibiting ferroptosis (Chen Z. et al., 2022). The islet cell autoantigen 69, which can regulate inflammation and immune response, induces ferroptosis to cause septic cardiac dysfunction through the stimulator of interferon gene trafficking (Kong et al., 2022). The neutrophil-derived lipocalin-2 induces ferroptosis by increasing the labile iron pool in the cardiomyocytes of LPS-induced mouse SCM model (Huang Q. et al., 2022).

## Pharmacological inhibition of ferroptosis for treating cardiomyopathy

Ferroptosis was first described in 2012; the studies on its role in cardiomyopathy are still in their infancy. However, existing evidence suggests a strong correlation between ferroptosis and cardiomyopathy. Thus, the inhibition of ferroptosis may be a promising target for treating cardiomyopathy. Ferroptosis reportedly plays a pathogenic role in cardiomyopathy; thus, scientists have begun identifying a targeted antiferroptosis approach for cardiomyopathy treatment. Numerous drugs have been recognized to exert a therapeutic impact on cardiomyopathy treatment by inhibiting ferroptosis. Several experimental compounds and clinical drugs inhibit ferroptosis to achieve therapeutic purposes in cardiomyopathies. The pharmacological inhibition of ferroptosis is becoming a cardioprotective strategy for cardiomyopathy prevention *in vitro* or *in vivo*. We try to sort these ferroptosis-inhibiting small molecules by mode of action. These categories maybe include activator of system Xc^−^, ferroptosis-inhibiting Nrf2 activators, GPX4 activator (direct or indirect), ferroptosis inhibitors through combined mechanisms, or ferroptosis inhibitors through unknown mechanisms. However, it is hard to clearly classify the ferroptosis-inhibiting small molecules into a specific categories.

### Inhibiting ferroptosis to treat ICM

Icariin **(1)** (Liu X. et al., 2021), xanthohumol **(2)** (Lin et al., 2022), britanin **(3) (** Lu et al., 2022), etomidate **(4) (** Lv et al., 2021),GAA**(5)** (Lin et al., 2021),dexmedetomidine **(6)** (Wang H. et al., 2022), sulforaphane **(7)** (Tian Y. et al., 2021), naringenin **(8)** (Xu et al., 2021), C3G **(9)** (Shan X. et al., 2021), resveratrol **(10) (** Xu et al., 2019; Li D. et al., 2022)**,**5-aza-CdR **(11)** (Li W. et al., 2021), ferulic acid **(12)** (Liu XJ. et al., 2021), PDA NPs **(13)** (Zhang H. et al., 2021), atorvastatin **(14) (** Peng et al., 2022), baicalin **(15) (** Fan et al., 2021), and ponatinib + deferoxamine**(16) (** Tu et al., 2021) alleviate ICM through inhibiting ferroptosis (Table 1).

**TABLE 1:** Emerging compounds targeting key regulators of ferroptosis to inhibit ischemic cardiomyopathy.

Compounds	Type	Experimental model	Findings	Mode of action	References
Icariin **(1)**	Flavonoid	HR/H9c2 cells	↑Cell viability; ↓oxidative stress; ↓lactate dehydrogenase content; ↓Fe^2+^; ↓ACSL4; ↑ GPX4; ↑Nrf2 and HO-1	Nrf2/GPX4	Liu et al. (2021a)
Xanthohumol **(2)**	Chalcone	IRI/SD rats	↓Myocardial infarct size; ↓*Ptgs2 and Acsl4*; ↑NRF2; ↑GPX4; ↓ACSL4	Nrf2/GPX4	Lin et al. (2022)
Xanthohumol **(2)**	Chalcone	HR/H9c2 cells	↑Cell viability; ↓lipid peroxidation and ROS; ↓Fe^2+^;↑NRF2; ↑GPX4	Nrf2/GPX4	Lin et al. (2022)
Britanin **(3)**	Sesquiterpene lactone	IRI/SD rats	↓Infarct area; ↓myocardial apoptosis; ↓CK; ↓LDH; ↓ferroptosis; ↑AMPK/GSK3β/Nrf2	Nrf2/GPX4	Lu et al. (2022)
Britanin **(3)**	Sesquiterpene lactone	HR/H9c2 cells	↑Cell viability; ↓LDH; ↑GPX4; ↑GSH; ↓ROS; ↓Fe^2+^; ↓MDA	Nrf2/GPX4	Lu et al. (2022)
Etomidate **(4)**	Anesthetic agent	IRI/SD rats	↓Cardiac dysfunction; ↓myocardium damage; ↓CK and LDH; ↓collagen II and α-smooth muscle actin; ↓inflammatory factors (IL-6, IL-1β, and TNF-α); ↑SOD content; ↑GSH; ↑GPX4; ↓MDA; ↓Fe^2+^; ↓ACSL4; ↑Nrf2 and HO-1	Nrf2/GPX4	Lv et al. (2021)
GAA **(5)**	Polyphenol	MIRI/rat	↓Infarct size; ↓HNE; ↓PTGS2; ↓ACSL4; ↓Nrf2; ↑GPX4	Nrf2/GPX4	Lin et al. (2021)
Dexmedetomidine **(6)**	Sedative agent	IRI/rats	↓Myocardial infarction; ↑heart function; ↓Fe^2+^ and LPO in cardiomyocytes; ↑Nrf2, SLC7A11, and GPX4	Nrf2/xCT/GPX4	Wang et al. (2022a)
Dexmedetomidine **(6)**	Sedative agent	HR/H9c2 cells	↑Cell viability; ↓Fe^2+^ and lipid peroxidation; ↑levels of GSH and SOD activity; ↑AMPK/GSK-3β/Nrf2	Nrf2/xCT/GPX4	Wang et al. (2022b)
Dexmedetomidine **(6)**	Sedative agent	IRI/rats	↓Myocardial injury; ↓mitochondrial dysfunction; ↓ROS; ↓mitochondrial dysfunction; ↑SLC7A11; ↑GPX4; ↓FTH; ↓COX-2	Nrf2/xCT/GPX4	Yu et al. (2022a)
Sulforaphane **(7)**	Dietary phytochemicals	Rat/HFD/STZ	↓Myocardial infarct size; ↓CK-MB and LDH; ↓protein levels of ACSL4; ↑Nrf2 and FPN1; ↑GPX4	Nrf2/xCT/GPX4	Tian et al. (2021a)
Sulforaphane **(7)**	Dietary phytochemicals	H9C2/high glucose	↑Cell viability; ↓protein levels of ACSL4; ↑Nrf2 and FPN1; ↑GPX4	Nrf2/xCT/GPX4	Tian et al. (2021b)
Naringenin **(8)**	Natural flavonoid	MIRI/SD rat	↓Pathological damage; ↓ inflammation and LPO; ↓LDH and CPK; ↑Nrf2; ↑SLC7A11; ↑GPX4; ↑FTH1; ↑FPN1; ↓ NOX1	Nrf2/xCT/GPX4/Fe^2+^	Xu et al. (2021)
Naringenin **(8)**	Natural flavonoid	OGD/R/H9c2 cells	↓ MDA and LPO; ↓Fe^2+^;↑GSH and SOD; ↑Nrf2; ↑SLC7A11; ↑GPX4; ↑FTH1; ↑FPN1; ↓ NOX1	Nrf2/xCT/GPX4/Fe^2+^	Xu et al. (2021)
C3G **(9)**	Anthocyanin	MIRI/rat	↓Infarction area; ↓pathological alterations; ↓ST segment elevation; ↓ferroptosis related protein expression; ↓oxidative stress; ↓USP19; ↓Beclin1; ↓NCOA4; ↓ LC3II/LC3I	GPX4/Fe^2+^	Shan et al. (2021a)
C3G **(9)**	Anthocyanin	OGD/R/H9c2 cell	↓Oxidative stress; ↓LC3II/LC3I; ↓ autophagosome number; ↓ TfR1; ↑FTH1 and GPX4	GPX4/Fe^2+^	Shan et al. (2021b)
Resveratrol **(10)**	Natural polyphenol	MIRI/rat	↓Oxidative stress; ↓Fe^2+^ content; ↑GPX4 and FTH1; ↑USP19-Beclin1 autophagy	GPX4/Fe^2+^	Li et al. (2022a)
Resveratrol **(10)**	Natural polyphenol	OGD/R/H9c2 cells	↓Oxidative stress; ↓Fe^2+^ content; ↑GPX4 and FTH1; ↑USP19-Beclin1 autophagy	GPX4/Fe^2+^	Li et al. (2022b)
5-aza-CdR **(11)**	DNMT-1 inhibitor	MIRI/rat	↓Oxidative stress; ↓Fe^2+^ content; ↑GPX4 and FTH1; ↓NCOA4 and DNMT-1; ↑Beclin1	GPX4/Fe^2+^	Li et al. (2021a)
5-aza-CdR **(11)**	DNMT-1 inhibitor	OGD/R/H9c2 cells	↓Oxidative stress; ↓Fe^2+^ content; ↑GPX4 and FTH1; ↓NCOA4 and DNMT-1; ↑Beclin1	GPX4/Fe^2+^	Li et al. (2021b)
Ferulic acid **(12)**	Natural phenolic antioxidant	MIRI/SD rat	↓Infarct size; ↓ST segment; ↓CK; ↓LDH; ↓NT-proBNP content; ↓Ptgs2 mRNA; ↓Fe^2+^; ↑GSH/GSSG; ↑SOD, CAT and GSH-Px; ↓MDA; ↓ROS; ↑generation of ATP; ↑AMPKα2 and GPX4	GPX4/Fe^2+^	Liu et al. (2021b)
PDA NPs **(13)**	Polydopamine	MIRI/mice	↓Infarct size; ↑cardiac functions; ↓MDA; ↓Fe^2+^ deposition; ↓LPO; ↑GPX4	GPX4	Zhang et al. (2021b)
Atorvastatin **(14)**	Statins	IRI/SD rats	↓Ferroptosis in I/R rat myocardium through the SMAD7/hepcidin pathway	Fe^2+^	Peng et al. (2022)
Atorvastatin **(14)**	Statins	HR/H9c2 cells	↑Cell viability; ↓mitochondrial shrinkage; ↓ROS; ↓Fe^2+^;↓FPN1; ↑SMAD7; ↓hepcidin	Fe^2+^	Peng et al. (2022)
Ponatinib + deferoxamine**(15)**	Iron ion chelator	IRI/SD rats	↓Myocardial infarct size; ↓CK; ↓ferroptosis	-	Tu et al. (2021)
Ponatinib + deferoxamine**(15)**	Iron ion chelator	HR/H9c2 cells	↓H/R injury (↓LDH release and necrosis percent); ↓ferroptosis	-	Tu et al. (2021)
Baicalin **(16)**	Natural flavonoid	MIRI/rat	↓ST segment elevation; ↓coronary flow (CF); ↓left ventricular systolic pressure; ↓ infarct area; ↓ pathological changes; ↓ lipid peroxidation; ↓ iron accumulation	LPO/Fe^2+^	Fan et al. (2021)
Baicalin**(16)**	Natural flavonoid	OGD/R/H9c2 cells	↓ Cell viability loss; ↓ lipid peroxidation; ↓ iron accumulation	LPO/Fe^2+^	Fan et al. (2021)
PDA NPs **(13)**	Polydopamine	OGD/R/H9c2 cells	↑Cells viability; ↓Fe^2+^ content; ↑mitochondrial functions	Fe^2+^	Zhang et al. (2021a)
GAA **(5)**	Polyphenol	Ferroptosis inducer/NRCM	↑Cells viability; ↓LPO	LPO	Lin et al. (2021)
GAA **(5)**	Polyphenol	Ferroptosis inducer/H9c2 cells	↑Cells viability; ↓Fe^2+^ content; ↓MDA; ↓ ROS; ↑PTGS2	Fe^2+^	Lin et al. (2021)
GAA **(5)**	Polyphenol	OGD/R//NRCM	↑Cells viability; ↓LPO	LPO	Lin et al. (2021)

ACSL4, acyl-CoA, synthetase long-chain family member 4;AMPK, adenosine monophosphate activated protein kinase; C3G, Cyanidin-3-Glucoside; CK, creatine kinase; DNMT-1, DNA (cytosine-5)-methyltransferase 1; FTH1, ferritin heavy chain 1; GAA, gossypol acetic acid; GPX4, glutathione peroxidase 4; GSK3β, glycogen synthase kinase 3β; HR, Hypoxia/reoxygenation; H_2_S, hydrogen sulfide; HR, Hypoxia/reoxygenation; LDH, lactate dehydrogenase; LPO, lipid peroxidation; NCOA4, nuclear receptor coactivator 4; NRCM, neonatal rat cardiomyocytes; Nrf2, nuclear factor erythroid 2-related factor 2; TfR1, transferrin receptor protein 1.

#### Activators of System Xc^-^

Icariin **(1)**, a natural flavonoid compound, is the main component of the Chinese herb Epimedium (also called YinYangHuo in Traditional Chinese Medicine) that has the functions of anti-aging, anti-inflammation, antioxidation, anti-osteoporosis, and ameliorating fibrosis (Su et al., 2023).**1** is a potent inducer of Nrf2 (Moratilla-Rivera et al., 2023). **1** inhibit hypoxia/reoxygenation (H/R)-induced ferroptosis by increasing GPX4 and decreasing ACSL4 and content of Fe^2+^ in cardiomyocytes through activating the Nrf2/HO-1 signaling pathway (Liu X. et al., 2021). Owing to outstanding medicinal properties in preventing and curing many common health issues, **1** and its derivates, icariside II (ICS) and icaritin (ICT) have garnered great interest in drug development. **1** possesses a variety of beneficial effects in regulating cardiovascular inflammation and other biological activities. In China, YinYangHuo and its compound have been used in the treatment of numerous diseases, like AD, stroke, and depression. ICA and its metabolites, which contain flavonoids, polysaccharides, vitamin C, and other active compounds, have been proven to have cardio-cerebrovascular protective benefits (Wang et al., 2023). **1** can works as a prodrug was subjected to preclinical studies. We must realize that the oral bioavailability rate is only 12.02% for **1**. Studies have shown the addition of cyclodextrins (CDs) to ICA can result in a vast increase in its water solubility, consequently achieving considerably better bioavailability (Cui et al., 2013; Jin et al., 2013). The degradation of ICA into ICS *in vivo* promotes ICA absorption (Cheng et al., 2015).

Xanthohumol **(2)** is a principal prenylated chalcone isolated from hops with its anti-inflammatory, anti-oxidative, and cancer-preventive properties (Zhou et al., 2021; Neumann et al., 2022; Vicente de Andrade Silva et al., 2023).2 protect cardiomyocytes against Fe-SP- and RSL3-induced ferroptosis by decreasing the production of lipid peroxidation and ROS, chelating iron, increasing the Nrf2 and GPX4 protein, while decreasing the mRNA levels of *Ptgs2* and *Acsl4*, and the protein levels of ACSL4 (Lin et al., 2022). The poor solubility and stability severely limit **2** utilization (Luo et al., 2023). Britanin **(3)** is a potent inducer of Nrf2(Wu et al., 2017). **3** exert cardioprotective effect against IR-mediated damage through inhibiting ferroptosis by activation of the AMPK/GSK3β/Nrf2 signalling thereby upregulating GPX4 (Lu et al., 2022). Etomidate **(4)** is an ultrashort-acting, non-barbiturate hypnotic intravenous anesthetic agent. **4** mitigated IR-induced ICM through inhibiting ferroptosis by upregulating GPX4 expression, and decreasing the levels of MDA and iron and ACSL4. Nrf2 inhibitors ML385 eliminated the inhibition of **4** on ferroptosis induced by IR, suggesting that **4** attenuated the myocardial injury by inhibiting IR-induced ferroptosis via Nrf2(Lv et al., 2021). Gossypol Acetic Acid (GAA, **5**), a natural product taken from the seeds of cotton plants, attenuates ICM through inhibiting ferroptosis by chelating iron content, and downregulating mRNA levels of Ptgs2 in RSL3, and Fe-SP-induced H9c2, inhibiting LPO in oxygen-glucose deprivation/reperfusion (OGD/R)-induced H9c2.**5** attenuates IR-induced ICM through inhibiting ferroptosis by decreasing the production of LPO, increasing the Nrf2 and GPX4 protein, while decreasing the mRNA levels of *Ptgs2* and *Acsl4*, and the protein levels of ACSL4 (Lin et al., 2021). Dexmedetomidine **(6)**, a highly selective alpha2-adrenoceptor agonist with sedative, analgesic, sympatholytic, and hemodynamic-stabilizing properties, posess the protective effect against I/R (Xiao Z. et al., 2021; Chen Y. et al., 2021; Deng et al., 2022; Yang et al., 2022; Hu et al., 2023) and H/R (Wu W. et al., 2022; Wang L. et al., 2022) induced cardiomyocyte injury. **6** attenuates ICM through inhibiting ferroptosis by activating AMPK/GSK-3β-dependant Nrf2/SLC7A11/GPX4 (Wang et al., 2022d).

Sulforaphane **(7)** is a naturally occurring dietary phytochemical extracted from cruciferous vegetables (Zheng et al., 2022).**7** is a potent Nrf2 activators and inhibit cardiomyopathy (Xin et al., 2018; Su et al., 2021; Wang et al., 2022e). **7** is an important member of the isothiocyanates, and is abundant in cruciferous plants with excellent anti-cancer effects (Wei LY. et al., 2022).**7** attenuates ICM in diabetic rats through inhibiting ferroptosis by activation of Nrf2/FPN1 pathway (Tian H. et al., 2021). As a well known activator of Nrf2, **7** can upregulate multiple antioxidants and protect against various oxidative damages. **7** prevents rat cardiomyocytes from H/R injury *in vitro* via activating SIRT1 and subsequently inhibiting ER stress (Li et al., 2016). **7** protects from myocardial ischemia-reperfusion damage through activating Nrf2 (Silva-Palacios et al., 2019).**7** inhibit intermittent hypoxia-induced cardiomyopathy in mice through activating Nrf2 (Zhou et al., 2018). Several clinical studies with 7 for the (supportive) treatment of non-alcoholic fatty liver disease (NCT04364360), chronic kidney disease (NCT05153174, NCT04608903) and anthracycline related cardiotoxicity in breast cancer (NCT03934905) are ongoing. A multi-center, randomized, placebo-controlled clinical trial is needed to be conducted to investigate **7** in adult patients with ICM.

The dietary natural polyphenolic flavonoid compounds are found in various citrus fruits, bergamots, tomatoes, and other fruits, naringenin **(8)** ameliorates myocardial injury and cardiac dysfunction (Yu et al., 2019; Ye et al., 2020; He et al., 2022; Sutanto et al., 2022). **8** alleviates ICM by regulating the Nrf2/SLC7A11/GPX4 axis to inhibit ferroptosis (Xu et al., 2021). C3G **(9)** (a natural anthocyanins) (Shan X. et al., 2021), resveratrol **(10)** (Xu et al., 2019; Li T. et al., 2022), 5-aza-CdR **(11)** (Li Y. et al., 2021), ferulic acid **(12)** (Liu XJ. et al., 2021), and polydopamine nanoparticles (PDA NPs, **13)** (Zhang H. et al., 2021) alleviate ICM through inhibiting ferroptosis by upregulating GPX4.

#### Ferroptosis-related compounds targeting iron

**13** (Zhang et al., 2021b),atorvastatin **(14)** (Peng et al., 2022),ponatinib/deferoxamine **(15)** ^[76]^, and baicalin **(16)** (Fan et al., 2021) are classified into this groups (Table 1).**13** functions as a new type of ferroptosis inhibitor through inhibiting Fe^2+^ accumulation and restoring mitochondrial functions in H9c2 cells and reduced Fe^2+^ deposition and lipid peroxidation in a myocardial I/R injury mouse model (Zhang H. et al., 2021). Atorvastatin **(14)** is a potent, orally available inhibitor of hepatic 3-hydroxy-3-methylglutaryl-coenzyme A (HMG-CoA) reductase, the major rate-limiting enzyme in cholesterol synthesis.**14** reversed erastin or H/R-induced cell injury in H9C2 cells through inhibiting ferroptosis by decreasing Fe^2+^ via upregulating expression of FPN1(Peng et al., 2022). **14** increased the expression of the SMAD7 and decreased the expression of the hepcidin in H/R-induced H9C2 cells (Peng et al., 2022).**14** protects myocardium against ischemia-reperfusion injury through various mechanisms (Zuo et al., 2016; Chen et al., 2017; Yang et al., 2018; Cao et al., 2019). Two clinical studies with **14** for the treatment of dilated cardiomyopathy (www.clinicaltrials.gov,NCT01015144) and hypertrophic cardiomyopathy (www.clinicaltrials.gov,NCT00317967) were completed ongoing. However, there is no clinical trials to study the effect of **14** on ICM, remaining an open conundrum for future investigate on.**15**, an iron chelators can block ferroptosis. The combination of ponatinib with **15** exerted synergistic effect on reducing H/R injury, showing simultaneous suppression of ferroptosis^[76]^.

### Inhibiting ferroptosis to treat DCM

6- Gingerol **(17)** (Wu X. et al., 2022), curcumin **(18)** (Wei J. et al., 2022), canagliflozin **(19)** (Du et al., 2022), L6H21 **(20)** (Sumneang et al., 2022), sulforaphane **(7)** (Wang Z. et al., 2022), and SR9009 **(21)** (Huang Q. et al., 2022) attenuate DCM through inhibiting ferroptosis (Table 2). The major active components of ginger **17** have protect cardioprotective effect (Zhang et al., 2019; Ma et al., 2021; Han et al., 2022a; Han et al., 2022b). **17** alleviates DCM through inhibiting ferroptosis by decreaseing the expression of FACL4 and the content of iron and enhancing the expression of Nrf2/GPX4 (Wu X. et al., 2022). **18**, a natural polyphenol phytochemical derived from turmeric with antioxidant, anti-inflammatory, and anticancer properties, activates Nrf2/HO-1 signaling to relieve diabetic cardiomyopathy injury (Ren et al., 2020; Wu et al., 2020; Wei et al., 2021; Wu S. et al., 2022).**18** alleviates DCM through inhibiting ferroptosis by activating Nrf2, leading to upregulating GPX4, highlighting a potentially new therapeutic route for investigation for the treatment DCM(Wei J. et al., 2022). **19** is an anti-diabetes drug belongs to sodium-glucose cotransporter 2 inhibitor with extra cardiovascular benefits (Zhang et al., 2023). **19** alleviates DCM through inhibiting ferroptosis by activating system Xc^−^/GSH/GPX4 axis and regulating iron homeostasis (Du et al., 2022). Myeloid differentiation factor 2 (MD2) inhibitor L6H21 **(20)** alleviates DCM through inhibiting ferroptosis in prediabetic rats (Sumneang et al., 2022). As a Rev-erbs agonist SR9009 **(21)** alleviates DCM through inhibiting ferroptosis through by down-regulating ferritinophagy (Huang Y. et al., 2022). Sulforaphane **(7)** also works as an activators of System Xc^−^ to alleviates DCM(Wang D. et al., 2022).

**TABLE 2:** Emerging compounds targeting key regulators of ferroptosis to inhibit diabetic cardiomyopathy.

Compounds	Type	Experimental model	Effects	Mode of action	References
6-Gingerol **(17)**	Natural Antioxidant	C57BL/6J mice/HFD/STZ	↓Cardiac injury; ↓cardiomyocyte hypertrophy and interstitial fibrosis; ↑heart function; ↓FACL4; ↓Fe^2+^;↑GPX4; ↓IL-1β, IL-6, and TNF-α; ↑Nrf2 pathway; ↑SOD; ↓MDA	Nrf2/GPX4	Wu et al. (2022a)
6-Gingerol **(17)**	Natural Antioxidant	Rat H9C2/high glucose	↓Cardiac injury; ↓FACL4; ↓Fe^2+^;↑GPX4; ↓IL-1β, IL-6, and TNF-α; ↑Nrf2; ↑SOD; ↓MDA	Nrf2/GPX4	Wu et al. (2022b)
Curcumin **(18)**	Natural polyphenol	Rabbits/Streptozotocin	↓Fibrosis and collagen expression; ↓ACSL4 and COX2; ↑Nrf2,GPX4	Nrf2/GPX4	Wei et al. (2022a)
Curcumin **(18)**	Natural polyphenol	Rat H9C2/high glucose	↑Nrf2,GPX4; ↓ROS	Nrf2/GPX4	Wei et al. (2022b)
Sulforaphane **(7)**	Dietary phytochemicals	C57BL/6J mice/HFD/STZ	↑ Activation of AMPK/Nrf2; ↑ferritin; ↑SLC7A11; ↑ GSH and GSH/GSSG; ↓Fe^2+^; ↓COX2; ↓MDA	Nrf2/SLC7A11/Fe^2+^	Wang et al. (2022c)
Sulforaphane **(7)**	Dietary phytochemicals	ECTs/AGE	↑Cell viability; ↑Nrf2; ↓COX2; ↓MDA; ↑ferritin; ↑SLC7A11; ↑ GSH and GSH/GSSG; ↓Fe^2+^; ↑activation of AMPK	Nrf2/SLC7A11/Fe^2+^	Wang et al. (2022d)
Canagliflozin **(19)**	Antidiabetic drug	C57BL/6 mice/STZ	↓Damage of cardiac function; ↓LDH; ↓cTnI; ↓ myocardial fiber breakage, inflammation, collagen fiber deposition and mitochondrial structural disorder; ↓PCO; ↓MDA; ↑SOD; ↑CAT; ↑GSH; ↓deposition of total iron and Fe^2+^; ↓FTH; ↑SLC7A11	Nrf2/xCT/Fe^2+^	Du et al. (2022)
Canagliflozin **(19)**	Antidiabetic drug	Rat H9C2/high glucose	↓ROS; ↓Lipid-ROS; ↑MM; ↓PCO; ↓MDA; ↑SOD; ↑CAT; ↑GSH; ↓deposition of total iron and Fe^2+^; ↓FTH; ↑SLC7A11	Nrf2/xCT/Fe^2+^	Du et al. (2022)
L6H21 **(20)**	MD2 inhibitor	C57BL/6 mice/HFD	↓Insulin resistance; ↓cardiac autonomic imbalance and LV dysfunction; ↓cardiac mitochondrial dysfunction; ↓oxidative stress and inflammation; ↓cardiac apoptosis and ferroptosis; ↓ACSL4; ↑GPX4	GPX4/ACSL4	Sumneang et al. (2022)
SR9009 **(21)**	Rev-erbs agonist	Rat/HFD/STZ	↓Myocardial injury; ↓ferritinophagy/ferroptosis-related proteins	-	Huang et al. (2022b)

CAT, catalase; cTnI, cardiac troponin I; FTH, ferritin heavy chain;GSH, glutathione; GSSG, oxidized glutathione; HFD, high-fat diet; LC3Ⅱ, microtubule associated protein 3 Ⅱ; L6H21, myeloid differentiation factor 2 (MD2) inhibitor; MDA, malondialdehyde; MMP, mitochondrial membrane potential; NCOA4, nuclear receptor coactivator 4; LDH, lactate dehydrogenase;PCO, protein carbonyl; SLC7A11, solute carrier family 7 member 11; SOD, superoxide dismutase; STZ, streptozotocin.

### Inhibiting ferroptosis to treat DICM

Compounds that alleviate DICM through inhibiting ferroptosis include huaier polysaccharide **(22)** (Ma X. et al., 2022), LCZ696 **(23)** (Liu X. et al., 2022), fisetin **(24)** (Li D. et al., 2021), salidroside **(25)** (Chen H. et al., 2022), resveratrol **(10)** (Yu P. et al., 2022), dexazoxane **(26)** (Zhang H. et al., 2021), melatonin**(27)** (Sun et al., 2022), EGCG**(28)** (He et al., 2021), 16d and 16e **(29)** (Xu et al., 2022), ethoxyquin **(30)** (Tadokoro et al., 2022), and 7j **(31)** (You et al., 2022) inhibit DICM through inhibiting ferroptosis (Table 3). **22** is a naturally occurring bioactive macromolecule, found in Huaier fungus and has been shown to exert antitumor and antimetastasis activity (Tian Y. et al., 2021; Gou et al., 2022).**22** attenuates DICM through inhibiting ferroptosis by upregulating GPX4, suggesting its a direct activators of GPX4 (Ma X. H. et al., 2022). **23** is a first-in-class angiotensin receptor neprilysin inhibitor that can attenuate DICM through inhibiting ferroptosis (Liu H. et al., 2022). **23** significantly attenuated DICM by decreasing lipid ROS and MDA and increasing GPX4 and GSH. **23** activate AKT leading to increase SIRT3 expression and deacetylated SOD2. SIRT3 knockdown and AKT inhibition reversed the protective effect of **23** in H9c2 cells, suggesting that **23** prevents DICM by inhibiting ferroptosis via AKT/SIRT3/SOD2 signaling pathway (Liu M. Z. et al., 2022). Fisetin **(24)**, a naturally occurring polyphenol that is frequently present in a variety of fruits and vegetables, exert cardioprotective effect against cardiomyopathy (Althunibat et al., 2019; ALTamimi et al., 2021).**24** attenuates DICM through inhibiting ferroptosis *in vivo* and *in vitro* by activating SIRT1/Nrf2 signaling pathway (Li J. Y. et al., 2021). **24** increases GPX4 and GSH, thereby reducing the MDA and lipid ROS levels. Moreover, **24** exerted its protective effect by increasing the SIRT1 expression and the Nrf2 mRNA and protein levels and its nuclear translocation, which resulted in the activation of its downstream genes such as *HO-1* and *FTH1*. Selective inhibition of SIRT1 reversed the protective effects of **24** in the H9c2 cells, suggesting **24** exerts its therapeutic effects againstDICM by inhibiting ferroptosis via SIRT1/Nrf2 signaling pathway activation (Li W. et al., 2021). Salidroside **(25)**, a glucoside of the phenylpropanoid tyrosol isolated from Rhodiola rosea L, is a natural bioactive compound with anti-oxidative, anti-inflammatory, and neuroprotective properties (Zhao et al., 2021; Jin et al., 2022). **25** has cardiovascular benefits against cardiomyopathy (Ni T. et al., 2021; Li Y. et al., 2021). **25** attenuates DICM through inhibiting ferroptosis *in vivo* and *in vitro* by limiting iron accumulation, restoring GPX4, and preventing LPO (Chen L. et al., 2022). AMPK inhibitor compound C reversed protective function of **25** against DICM, suggesting that **25** markedly downregulated ferroptosis by activating AMPK-dependent signaling pathways (Chen Z. et al., 2022). Dexazoxane**(26)** (Zhang Y. et al., 2021), melatonin**(27)** (Sun et al., 2022), and EGCG**(28)** (He et al., 2021) also works as a GPX4 activators to attenuates DICM through inhibiting ferroptosis.

**TABLE 3:** Emerging compounds targeting ferroptosis to inhibit DOX-induced cardiomyopathy.

Compounds	Type	Experimental model	Effects	Mode of action	Reference
Huaier Polysaccharide **(22)**	Polysaccharide	DOX/BALB/c mice	↓cTnI and lactate dehydrogenase; ↓myocardial fibrosis; ↑GPX4	GPX4	Ma et al. (2022a)
LCZ696 **(23)**	Angiotensin receptor neprilysin inhibitor	DOX/H9c2 cell	↓lipid reactive oxygen species; ↓malondialdehyde; ↑GPX4; ↑GSH in cells and heart tissues; ↑SIRT3 expression and deacetylated its target gene SOD2	GPX4	Liu et al. (2022a)
LCZ696 **(23)**	Angiotensin receptor neprilysin inhibitor	DOX/rat	↑Remodeled myocardial structures; ↑heart ventricular function; ↑GPX4; ↑ GSH in cells and heart tissues	GPX4	Liu et al. (2022b)
Fisetin **(24)**	Flavonol	DOX/rat	↓Cardiac dysfunction; ↓cardiac hypertrophy; ↓myocardial fibrosis; ↑GPX4; ↓MDA; ↓lipid ROS; ↑GSH; ↓Fe^2+^; ↑Nrf2; ↓Keap1; ↑HO-1; ↑FTH1; ↓FPN; ↑TfR1	Nrf2/GPX4/Fe^2+^	Li et al. (2021c)
Fisetin **(24)**	Flavonol	DOX/H9c2 cell	↑GPX4 level; ↑SIRT1; ↑Nrf2 activation; ↑HO-1 and FTH1; ↓Fe^2+^	Nrf2/GPX4/Fe^2+^	Li et al. (2021d)
Salidroside **(25)**	Glucoside	DOX/Male C57/BL mice	↓Cardiac dysfunction; ↓cell damage; ↓fibrosis; ↓Fe^2+^; ↑GPX4; ↓LPO (↓MDA+4-HNE); ↓ROS; ↑MMP; ↑mitochondrial biogenesis; ↑mitochondrial iron-sulfur clusters; ↑mitochondrial OXPHOS complexes; ↑mitochondrial function; ↑AMPK	GPX4/Fe^2+^/LPO	Chen et al. (2022a)
Salidroside **(25)**	Glucoside	DOX/H9c2 cell	↓Fe^2+^;↑GPX4; ↓4-HNE; ↑AMPK	GPX4/Fe^2+^	Chen et al. (2022b)
Dexazoxane **(26)**	Iron chelator	DOX/Male Wistar rats	↓Cardiac dysfunction; ↓*PTGS2* mRNA and protein; ↓HMGB1	GPX4/Fe^2+^	Zhang et al. (2021a)
Dexazoxane **(26)**	Iron chelator	DOX/H9c2 cell	↑Cell viability; ↑GPX4 and FTH1; ↓MDA; ↓LDH	GPX4/Fe^2+^	Zhang et al. (2021b)
Melatonin **(27)**	Phytohormones	DOX/H9c2 cell	↑Cell viability; ↓mitochondrial dysfunction; ↓ACSL4; ↑ GPX4; ↑GSH-PX; ↑ p-YAP; ↓YAP	GPX4/ACSL4	Sun et al. (2022)
Melatonin **(27)**	Phytohormones	DOX/rat	↓Myocardial injury; ↓mitochondrial dysfunction; ↓cardiomyocyte size; ↓cardiac collagen fraction	GPX4/ACSL4	Sun et al. (2022)
Melatonin **(27)**	Phytohormones	DOX/zebrafish	↓Pericardial edemar; ↑ heart rate↓ACSL4; ↑ GPX4	GPX4/ACSL4	Sun et al. (2022)
EGCG **(28)**	Polyphenol	DOX/H9c2 cell	↑Cell viability; ↓LDH; ↓PTGS2; ↑GPX4; ↑AMPKα2 and promoted TCA cycle activation; ↓MDA; ↓4-HNE; ↑GSH; ↑GSH/GSSG; ↓ROS; ↓iron accumulation; ↓oxidative stress; ↓ipid metabolism	GPX4/LPO	He et al. (2021)
EGCG **(28)**	Polyphenol	DOX/C57BL/6 mice	↓CK-MB and LDH activity; ↑left ventricular function; ↓morphological myocardial changes; ↓PTGS2; ↑GPX4; ↑AMPKα2 and promoted TCA cycle activation	GPX4/LPO	He et al. (2021)
Resveratrol **(10)**	Natural polyphenol	DOX/H9c2 cell	↑Cell viability; ↓iron accumulation; ↓LPO; ↓mitochondrial ROS; ↑p62-Nrf2/HO-1	Nrf2/LPO	Yu et al. (2022a)
Resveratrol **(10)**	Natural polyphenol	DOX/mice	↑ Left ventricular function; ↓myocardial fibrosis; ↑ p62-Nrf2/HO-1; ↓ferroptosis	Nrf2	Yu et al. (2022b)
16d and 16e **(29)**	Steviol derivatives	DOX/zebrafish	↓Cardiac dysfunction; ↓ferroptosis; ↓GSH depletion; ↓iron accumulation; ↓LPO; ↓ROS; ↑MMP	LPO	Xu et al. (2022)
Ethoxyquin **(30)**	Radical-Trapping Antioxidant	DOX/Mice	↓Cardiac impairments; ↓serum LDH and CK; ↓MDA and acrolein; ↓cardiac fibrosis; ↓ TUNEL-positive cells	LPO	Tadokoro et al. (2022)
Ethoxyquin **(30)**	Radical-Trapping Antioxidant	DOX/Cardiomyocytes	↓Cell death; ↓ferroptosis; ↓MDA and mitochondrial lipid peroxides	LPO	Tadokoro et al. (2022)
7j **(31)**	Phenothiazine derivatives	DOX/C57BJ/6 mice	↓Fibrosis; ↓serum ALT; ↓serum AST; ↓serum CK; ↓serum LDH	-	You et al. (2022)

ALT, Alanine aminotransferase;AST, Aspartate aminotransferase;GSH, glutathione; GSSG, oxidized glutathione; Keap1, Kelch-like ECH-associated protein 1; MDA, malondialdehyde; SLC7A11, solute carrier family 7 member 11; FTH, ferritin heavy chain; NCOA4, nuclear receptor coactivator 4; MMP, mitochondrial membrane potential; MDA, malondialdehyde; SOD, superoxide dismutase; Nrf2, nuclear factor erythroid 2-related factor 2; ROS, reactive oxygen species.

While compounds 16d and 16e **(29)** (Xu et al., 2022), ethoxyquin **(30)** (Tadokoro et al., 2022), and resveratrol **(10)** (Yu P. et al., 2022) functions as inhibitors of LPO to attenuates DICM through inhibiting ferroptosis. **29** is two derivatives of steviol, an ent-kaurene diterpenoid, possesses broad-spectrum bioactivity. **29** attenuates DICM in zebrafish cardiomyopathy model through inhibiting ferroptosis via suppressing the GSH depletion, iron accumulation, and LPO, decreasing ROS overproduction, and restoring the mitochondrial membrane potential (Xu et al., 2022). Ethoxyquin (6-ethoxy-1,2-dihydro-2,2,4-trimethylquinoline, **30** is a competent radical-trapping antioxidant. **30** effectively prevented GPX4-deficient ferroptosis in cultured cardiomyocytes, accompanied by the suppression of MDA and mitochondrial lipid peroxides (Tadokoro et al., 2022). **30** ameliorated DICM *in vivo* through decreasing the levels of lipid peroxides such as MDA and acrolein (Tadokoro et al., 2022). **10** works as a potent p62 activator has potential as a therapeutic target in preventing DICM via inhibiting ferroptosis (Yu W. et al., 2022). The 2-vinyl-10H-phenothiazine derivatives 7j **(31)** is a new class of ferroptosis inhibitors, maintaining high ferroptosis inhibitory activity (EC50 = 0.001 µM on the erastin-induced HT1080 cell ferroptosis model) (You et al., 2022). **31** acted as a ROS scavenger displayed favorable pharmacokinetic properties and exhibited no obvious toxicity *in vivo* and vitro and could relieve DICM, providing a promising lead compound for drug discovery targeting ferroptosis to treat DICM(You et al., 2022).

### Inhibiting ferroptosis to treat SCM

Compounds that alleviate SCM through inhibiting ferroptosis include vitamin B6 **(32)** (Shan M. et al., 2021), ferrostatin-1 **(33)** (Xiao Y. et al., 2021), puerarin **(34)** (Zhou et al., 2022), H_2_S (NaHS) **(35)** (Cao et al., 2022), dexmedetomidine **(6)** (Wang et al., 2020), resveratrol **(10)** (Wang H. et al., 2022; Zeng et al., 2023), and attenuate SCM through inhibiting ferroptosis (Table 4).**10** alleviate SCM through inhibiting ferroptosis via decreasing LPO, and increasing Sirt1/Nrf2 expression. EX527, a selective Sirt1 inhibitor reversed the protective effect of **10** against ferroptosis (Zeng et al., 2023). This observation was corroborated by other study, which reported **10** upregulated miR-149 and downregulated HMGB1 to inhibit ferroptosis and improve SCM(Wang L. et al., 2022). Vitamin B6 **(32)** can suppress LPS-induced oxidative stress and LPO that lead to ferroptosis *in vivo* and *in vitro* through activating Nrf2. **32** can regulate the expression of iron regulatory proteins, maintaining intracellular iron homeostasis (Shan M. et al., 2021). Ferrostatin-1 **(33)**, a ferroptosis inhibitor, improves SCM through inhibiting ferroptosis (Xiao Y. et al., 2021).**33** alleviate SCM through inhibiting ferroptosis via decreasing LPO, PTGS2, ferritin light chain (FTL) and ferritin heavy chain (FTH1), while upregulating GPX4 and ferroportin (FPN, SLC40A1) (Xiao Z. et al., 2021). Compounds puerarin **(34)** is an isoflavone compound derived from *Pueraria lobata* in traditional Chinese medicine with cardiovascular benefits against cardiomyopathy (Qin et al., 2016; Li et al., 2017; Yin et al., 2019; Wang et al., 2022d). **34** inhibite SCM induced by LPS through inhibiting ferroptosis via upregulating GPX4 and ferritin and downregulating ACSL4, TfR, and iron content (Zhou et al., 2022).

**TABLE 4:** Emerging compounds targeting ferroptosis to inhibit sepsis-induced cardiomyopathy.

Compounds	Type	Experimental model	Effects	Mode of action	Reference
Resveratrol **(10)**	Natural polyphenol	CLP/rats	↓Cardiac dysfunction; ↓myocardial damage; ↓impaired mitochondria; ↓lipid peroxidation; ↑Sirt1/Nrf2	Nrf2	Zeng et al. (2023)
Resveratrol **(10)**	Natural polyphenol	LPS/mice	↑Cardiac function; ↓cardiomyocyte injury	Nrf2	Wang et al. (2022f)
Vitamin B6 **(32)**	Vitamin	LPS/C57BL/6 mice	↓Myocardial injury; ↓oxidative stress; ↓lipid peroxidation; ↓MDA; ↑SOD; ↑GSH; ↑Nrf2	Nrf2	Shan et al. (2021a)
Vitamin B6 **(32)**	Vitamin	LPS/rat H9c2 cardiomyocytes	↓MDA; ↑SOD; ↓lipid peroxidation; ↓TFR; ↓ferritin; ↑FPN1; ↑Nrf2; ↑GPX4; ↑HO-1; ↑NQO1	Nrf2/GPX4/Fe^2+^	Shan et al. (2021b)
Dexmedetomidine **(6)**	Sedative agent	CLP/rats	↓Myocardial injury; ↓MDA; ↓8-hydroxy-2′-deoxyguanosine; ↓IL-6 and monocyte chemoattractant protein-1; ↑GPX4, SOD and GSH; ↓HO-1; ↓TfR; ↓cleaved caspase 3; ↓inducible nitric oxide synthase; ↓ gasdermin D; ↓iron concentration	GPX4/Fe^2+^/LPO	Wang et al. (2020)
Ferrostatin-1 **(33)**	Ferroptosis antagonist	LPS/rat	↑Cardiac systolic function; ↓cardiac injury markers; ↓PTGS2; ↓iron deposition in the myocardium; ↑ferroportin (FPN, SLC40A1); ↓FTL; ↓FTH1 expression; ↓lipid peroxidation; ↑GPX4; ↓TNF-α, IL-1β, and IL-6; ↓TLR4, phospho-NF-κB, and phospho-IκBα	GPX4/Fe^2+^	Xiao et al. (2021b)
Puerarin **(34)**	Isoflavone	LPS/SD rat	↓Myocardial injury; ↑GPX4 and ferritin; ↓ACSL4, TFR, and heart iron content	GPX4/Fe^2+^/ACSL4	Zhou et al. (2022)
NaHS **(35)**	H_2_S	CLP/rats	↓Septic myocardial ferroptosis; ↑cardiac dysfunction; ↓myocardial cell and tissue injury; ↓phosphorylation of BECN1; ↑expressions of SLC7A11 and GPX4	SLC7A11/GPX4	Cao et al. (2022)
Resveratrol **(10)**	Natural polyphenol	LPS/ventricular tissues	↑Cardiomyocyte viability; ↑GSH; ↓ LDH secretion; ↓lipid ROS; ↓LPO; ↓iron accumulation	LPO/Fe^2+^	Wang et al. (2022e)
NaHS **(35)**	H_2_S	LPS/rat H9c2 cardiomyocytes	↑Cell viability; ↓ferroptosis; ↓Fe^2+^;↓oxidative stress	Fe^2+^	Cao et al. (2022)

CK-MB, Creatine Kinase MB; CLP, cecal ligation and puncture; FPN, ferroportin (SLC40A1); FTL, ferritin light chain;FTH, ferritin heavy chain; GSH, glutathione; GSSG, oxidized glutathione; HO-1, heme oxygenase-1; IL-1β, interleukin-1; IκBα, inhibitor of kappa Bα; LDH, lactate dehydrogenase;LPO, lipid peroxidation; LPS, lipopolysaccharide; MDA, malondialdehyde; NCOA4, nuclear receptor coactivator 4; NF-κB, nuclear factor kappa B; PTGS2,prostaglandin endoperoxide synthase 2; SLC7A11, solute carrier family 7 member 11; SOD, superoxide dismutase; TfR, transferrin receptor; TLR4,toll-like receptor 4; TNF-α, tumor necrosis-alpha.

## Conclusions and perspectives

The pathophysiology of cardiomyopathies is complex and still undergoing extensive investigation. In this review, we appraised articles that emphasized research progress in the pathological roles of ferroptosis in ICM, DCM, DICM, and SCM and ferroptosis inhibitors to mitigate cardiomyopathies. Meanwhile, researchers have identified novel targeted treatments for these cardiomyopathies through the pharmacological inhibition of ferroptosis. The pharmacological inhibition of ferroptosis is a potential therapeutic target for these cardiomyopathies, with potential novel drug targets and strategies for these diseases. However, the current research on the role of ferroptosis in cardiomyopathies is still in the infancy, and is still poorly understood. And more studies are required to clarify its role and functional mechanisms. Furthermore, most data reported in the literature are derived from experimental studies that do not directly report clinical applications and implications. Although a phase III clinical trial is underway to determine if resveratrol exert the potential heart benefits of resveratrol in patients with non-ischemic cardiomyopathy (phase III, n = 40, NCT01914081). In addition, a multi-center, randomized, placebo-controlled phase II clinical trial is also being conducted to investigate the LCZ696 in adult patients with non-obstructive hypertrophic cardiomyopathy (nHCM) (phase II, n = 45, NCT04164732). However, there is still lacking the study directly targeting ferroptosis to treat ICM, DCM, DICM, and SCM using bioactive compounds. Therefore, more clinical studies need to be conducted to inform practical treatment and management strategies. Despite these considerations, the current evidence strongly indicates that inhibiting ferroptosis marks a significant new direction for treating cardiomyopathies.
